# TRAIT AND STATE SUBJECTIVE AGING INTERACT TO PREDICT DAILY CONTROL BELIEFS

**DOI:** 10.1093/geroni/igz038.203

**Published:** 2019-11-08

**Authors:** Shenghao Zhang, Shevaun D Neupert

**Affiliations:** North Carolina State University, Raleigh, North Carolina, United States

## Abstract

We examine trait and state subjective aging as antecedents of control beliefs in older adults with a daily diary design. Adults (n=116) ranging in age from 60 to 90 (M=64.71) completed a nine-day daily diary study online. Participants reported trait aging attitudes (Attitudes Towards Own Aging; ATOA) on Day 1 and daily Awareness of Age-Related Change (AARC) of loss and gain experiences and control beliefs (Locus of Control and Perceived Competence) on Days 2-9. Controlling for demographics and known antecedents of control beliefs (health, stressors, emotional well-being, and cognition), daily increases in AARC gain were associated with increases in both Locus of Control and Perceived Competence, and a cross-level interaction revealed that Locus of Control decreased for those with more positive ATOA on days when they reported more AARC losses. Discussion will focus on interpreting the interaction between trait and state subjective aging.

